# Free-Weight Resistance Training Enhances Core Muscle Strength but Does Not Translate to Improved Athletic Performance in Adolescent Canoe/Kayak Athletes

**DOI:** 10.3390/children11101177

**Published:** 2024-09-27

**Authors:** Ting-Ting Lee, Bo-Jen Ko, Chu-Han Chang, I-Shiung Cheng

**Affiliations:** 1 Department of Aquatics Sports, University of Taipei, Taipei 10608, Taiwan; tingtinglee@go.utaipei.edu.tw; 2 Department of Physical Education, National Taichung University of Education, Taichung City 40306, Taiwan; kobojen@mail.ntcu.edu.tw; 3 Department of Sport Performance, National Taiwan University of Sport, Taichung City 404401, Taiwan; hwpigpie8@mail3.hwsh.tc.edu.tw

**Keywords:** strength, sprint kayak, water sports

## Abstract

Background/Objectives: While previous evidence has shown that using free weights for resistance training is a more practical approach to enhancing strength, there is a relatively low prevalence of free-weight resistance training among adolescent kayak/canoe athletes. Therefore, this study aims to assess the impact of free-weight resistance training on body composition and various performance factors among adolescent canoe/kayak athletes. Methods: Twenty-seven young sprint kayakers and canoeists (14 ± 1 years; 164 ± 7 cm; 56 ± 8 kg) completed this study. Following baseline assessments, athletes were randomly divided into two training groups: the free-weight resistance training group (FW) or the control group (C). The FW group underwent free-weight resistance training sessions twice weekly for 24 weeks. The C group maintained their regular bodyweight training sessions during the same timeframe. All participants performed both the pre- and post-training assessments for the following dependent variables: body composition, upper-body power, upper-body isometric muscle strength, isometric mid-thigh pull, core strength, countermovement jump, balance, anaerobic ability, and aerobic performance. Results: After 24 weeks of training, the free-weight resistance training group exhibited a significant increase in body weight (from 56 ± 5 kg to 58 ± 4 kg, *p* < 0.05) and improvements in the number of straight leg raise repetitions (from 23 ± 6 to 26 ± 4, *p* < 0.05) compared to the control group. However, the two groups observed no significant differences between upper-body isometric muscle strength, power, balance, and anaerobic/aerobic performance. Conclusions: A 24-week training duration might be insufficient for novice participants in resistance training. Future research should consider incorporating an adaptation period or a learning phase for movements before training, thereby enhancing the efficacy of free-weight resistance training in increasing strength.

## 1. Introduction

Flat-water sprint kayaking, an Olympic sport since 1936, features race distances of 200, 500, and 1000 m, with times ranging from about 35 s to 4 min. The contribution of energy systems varies significantly by distance: at 200 m, aerobic and anaerobic systems contribute 37% and 63%, respectively, whereas at 1000 m, the contributions shift to 82% aerobic and 18% anaerobic [1]. Gabler et al. [2] evaluated adolescent kayakers and canoeists using various physical fitness tests and found that performance in 2-min bench pull and bench press muscular endurance tests was highly positively correlated with 500 m and 2000 m race times on the water (R^2^_500m_ = 0.77; R^2^_2000m_ = 0.64, 95% confidence interval). Kristiansen et al. [3] conducted a 6-week strength training intervention to increase the one-repetition maximum (1RM) in bench press. The training group showed a significant increase in 1RM strength in bench press, from 87.3 ± 21.2 kg before the intervention to 93.9 ± 21.3 kg after (*p* = 0.001), and in bench pull, from 84.2 ± 15.3 kg to 86.0 ± 15.1 kg (*p* = 0.025). The group also significantly reduced the time to complete the 200 m kayak ergometer sprint test, from 44.8 ± 4.3 s to 44.3 ± 4.3 s (*p* = 0.042). The study identified the 1RM bench press as the best predictor of 200 m kayaking performance; improvements in the 1RM bench press correlated with enhanced kayaking performance. Since kayaking propulsion relies on each stroke’s force, muscle strength crucially influences performance, making strength training essential for optimizing flat-water sprint kayaking [4].

With advancements in strength and conditioning training, safely setting intensity and volume for athletes is a primary concern for coaches. In grassroots adolescent training, coaches face significant challenges in planning due to past debates against adolescent resistance training. In the 1970s and 1980s, resistance training was rarely recommended for children and adolescents due to concerns over high injury risks. This apprehension was primarily based on data from the National Electronic Injury Surveillance System (NEISS) of the U.S. Consumer Product Safety Commission [5], aggregating injury data from various emergency departments to estimate nationwide exercise-related injuries. However, it is critical to understand that NEISS data reflects injuries attributed by patients to resistance training and equipment, making it inaccurate to conclude that these injuries were solely caused by such activities [6]. However, with the advancement of scientific training, further investigations have found that many injuries occur due to incorrect training techniques or accidents that happen without proper supervision. Previous studies have indicated that resistance training performed with rigorous design and professional monitoring of intensity and training volume does not hinder the growth and development of adolescents [7]. Furthermore, it can enhance overall muscle strength and improve adolescent athletic performance [8,9]. Additionally, it can effectively prevent and reduce the risk of athletic injuries among athletes [10,11,12]. Pichardo et al. [13] conducted a 28-week training program with resistance training and resistance training combined with weightlifting for 59 male athletes aged 12–14 years. Both groups experienced minor-to-moderate improvements in resistance training skills battery quotient (RTSQ), absolute isometric mid-thigh pull peak force (IMTP_ABS_), and ratio-scaled isometric mid-thigh pull peak force (IMTP_REL_) after the initial 14 weeks of training (effect size: d = 0.45–0.86). Additionally, after the subsequent 14 weeks of training, there were small-to-moderate improvements in lower body power, upper-body power, and speed (effect size: d = 0.30–0.95).

Free weight training involves using unrestricted loads in space, such as the barbell back squat, not affixed to another supporting structure [14]. Utilizing free weights for resistance training is a more practical approach to enhancing strength. This allows for extensive compound movements with decreased stability, leading to increased engagement of stabilizing muscles around the primary muscle groups. Additionally, it provides for a superior replication of sporting actions [12]. Furthermore, free-weight resistance training can increase muscle strength through multi-joint movements, which is crucial for many sports. Despite its technical complexity, it can be a safe and effective method to improve athletic qualities in young athletes [14]. Free weight training requires more stability and balance, which may increase muscle involvement [15], potentially assisting greatly with the balance and core stability required in kayaking sports. Past research has also indicated that increased muscle involvement during free weight activities may lead to more significant release of muscle growth-promoting hormones, such as free testosterone and growth hormone [16]. These hormones can stimulate muscle growth, resulting in more substantial muscle hypertrophy and strength gains [17]. However, little research was found regarding free weight resistance training in canoe/kayak training and evaluating its effectiveness on performance.

Previous scholars attempted to establish various training models based on the developmental stages of adolescent athletes. Cote [18] suggested the Long-Term Athlete Development (LTAD) model outlining three distinct phases: sampling years (ages 6–12), specializing years (ages 13–15), and investment years (ages 16 and above). However, some studies argue that categorizing athletes’ maturity status by age might not be appropriate as individuals of the same chronological age may vary significantly in biological age [19]. Biological age should be defined as an individual’s growth stage in terms of skeletal, gender, or constitutional factors [20]. The LTAD model proposed by Bayli and Hamilton involves longitudinal monitoring of changes in the physique, focusing on the timing and velocity of individual growth, including peak height velocity (PHV), the stage of maximum skeletal growth rate, and peak weight velocity (PWV), the stage of maximum bone and muscle mass accumulation associated with maturation. It recommends that coaches tailor training programs based on the athlete’s growth patterns [21]. However, in adolescent athletes, high muscle strength and power levels are crucial for their athletic performance [22]. In particular, methods of muscle strength training for adolescent canoe/kayak athletes are still rare in current research.

In summary, few studies compare the benefits of free-weight and bodyweight resistance training on adolescent sports performance. More studies are needed to investigate the effects of free-weight resistance training on various potential performance factors among adolescent canoe/kayak athletes. This study aims to conduct a 24-week free-weight resistance training intervention designed in collaboration with strength and conditioning experts to cater to adolescents’ growth and competition demands through a controlled experimental design. Thid study aims to observe the effects of free-weight resistance training on body composition and various performance factors through athletic performance assessments among adolescent canoe/kayak athletes.

## 2. Methods

### 2.1. Experimental Approach to the Problem

A controlled experimental trial was conducted to investigate the impact of free-weight resistance training on body composition and athletic performance among young canoe/kayak athletes aged 12–14 years. Following baseline assessments, athletes were randomly divided into two training groups: the free-weight resistance training group (FW) or the control group (C). The FW group underwent free-weight resistance training sessions twice a week for 24 weeks, each lasting 90 min, with progressively increasing intensity ranging from 60% to 80% of their 1RM. These sessions were conducted under the supervision of trainers certified by the National Strength and Conditioning Association (NSCA) as Certified Strength and Conditioning Specialists (CSCS). Meanwhile, the C group continued their regular bodyweight training sessions over the same period. The primary exercises included in their routine were running, push-ups, planks, and burpees, performed in a circuit training format. Participants whose attendance rates fell below 80% of the total training sessions were excluded from this study. All participants performed both the pre- and post-training assessments for the following dependent variables: body composition, upper-body power, upper-body isometric muscle strength (bench pull and bench press), isometric mid-thigh pull (IMTP), core strength, countermovement jump (CMJ), balance, anaerobic ability, and aerobic performance.

### 2.2. Participants

**Figure 1:**
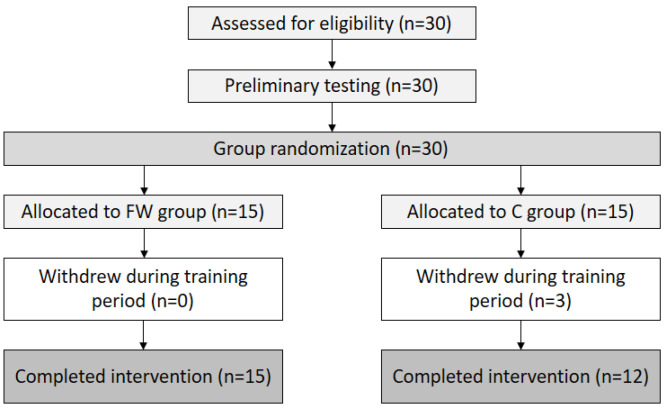
Flow chart of the participant’s progress from eligibility assessment to intervention completion.

Thirty semi-elite young sprint kayakers and canoeists voluntarily participated in this study, with twenty-seven (15 boys and 12 girls; aged 13.96 ± 1.37 years; height: 163.50 ± 6.89 cm; weight: 55.96 ± 8.27 kg) completing this study (Figure 1). All athletes were from the same local team and had undergone canoe/kayak training for at least one year, training more than thrice weekly over the past three months. Participants with any bone, muscle, or joint injury that would preclude them from performing the experimental sessions were excluded from this study. During the experimental period, participants attended a kayak training camp where they received the same water-based training program from a coach and the strength and conditioning training designed for this study. Training volume ranged from 12 to 28 h per week. Athletes who had excessive absences during the training period were excluded. Before participating in this study, subjects provided informed consent after being briefed on this study’s objectives and experimental protocol by the researchers. The study protocol was approved by the University of Taipei Institutional Review Board (UT-IRB No. 2022-058). The data collection period was from November 2022 to June 2023.

### 2.3. Procedure

#### 2.3.1. Body Composition

The InBody 270 body composition analyzer (Biospace Inc., West Des Moines, IA, USA) was utilized to measure body weight, skeletal muscle mass (SMM; kg), body fat mass (BFM; kg), and percent body fat (PBF). This analyzer operates based on three core principles: an eight-point tactile electrode system, segmental bioelectrical impedance analysis, and multifrequency bioelectrical impedance analysis. Before the measurement, participants were instructed to remove any metal objects they were carrying, assume a steady stance on a sensory surface for bodyweight measurement, input their basic information, and grasp a sensory console for additional measurement parameters. 

#### 2.3.2. Upper-Body Power

Three medicine ball throw tests were conducted to evaluate upper-body power: the chest push, overhead front throw, and overhead back throw. Participants stood in a standing position behind a marked line on the floor. Using both hands, they threw a 4 kg medicine ball from chest level or overhead, aiming for maximum horizontal distance. Participants were instructed to avoid using their lower body for power generation and stepping over the line after releasing the medicine ball. Each participant performed three trials, with the longest throw recorded for subsequent analysis. A 30 s rest period was observed between trials.

#### 2.3.3. Upper-Body Isometric Muscle Strength

The isometric seal row (ISR) and isometric bench press (IBP) were conducted using two PASCO PASPort Force Platforms (PS-2141, Wiltronics Research Pty. Ltd., Alfredton, Australia). Participants underwent team warm-up and one trial pull before the test. The Force Platforms were positioned above the bench. During testing, the height of the bar was adjusted according to each participant’s height, and the bar was securely fixed in place. During the ILR, participants assumed a prone position, gripping the bar with both hands while maintaining a 90-degree elbow angle. They kept their chest elevated, back straight, eyes directed downward, legs naturally extended, and heels not touching the edge of the rack to avoid stabilization attempts. During the bench press, participants laid flat on their backs, gripping the bar with both hands while maintaining a 90-degree elbow angle. They kept their back flat against the bench, their eyes directed upward, their feet naturally resting on the ground, and their toes not touching the edge of the bench to avoid stabilization attempts. During testing, upon hearing the command “ready”, participants lifted the bar to a system weight of approximately 100 N and held it steadily for 3 s. Upon hearing the command “3, 2, 1, PULL”, they quickly pulled or pushed the bar with maximum force, maintaining the maximum effort for five seconds, completing one trial. The peak force and rate of force development (RFD) were calculated for formal testing based on the average values of two full-force pulls or pushes. If the values of the two trials were less than 200 N, a third trial was conducted, and the best two values exceeding 200 N were selected for analysis.

#### 2.3.4. Isometric Mid-Thigh Pull (IMTP)

The Isometric Mid-Thigh Pull (IMTP) was performed using two PASCO PASPort Force Platforms (PS-2141, Wiltronics Research Pty. Ltd., Alfredton, Australia) based on the protocol referenced by Kaçoğlu et al. [23]. Participants underwent team warm-up and one trial pull before the test. During testing, the height of the bar was adjusted according to each participant’s height, and the bar was securely fixed in place. Participants began the movement with straight arms and grasped the bar with both hands using assistance straps. They maintained an upright posture, eyes facing forward, feet shoulder-width apart, and knees flexed at an angle of 125 to 135 degrees. During testing, upon hearing the command “ready”, participants lifted the bar to a system weight of approximately 200 N and held it steadily for 3 s. Upon hearing the command “3, 2, 1, PULL”, they quickly pulled the bar upwards with maximum force, maintaining the maximum effort for five seconds, completing one trial. The peak force and rate of force development (RFD) were calculated for formal testing based on the average values of two full-force pulls. If the values of the two trials were less than 500 N, a third trial was conducted, and the best two values exceeding 500 N were selected for analysis.

#### 2.3.5. Core Strength

This study used the Straight Leg Raise test (SLR) to assess the participants’ core strength. Participants were instructed to lie flat on the ground, with their hands holding a support overhead and their legs fully extended. They were then required to raise their legs upward as far as possible, ensuring that the legs remained straight and surpassed a 90-degree angle with the torso, completing one repetition of the straight leg raise. The test protocol involved participants attempting to perform as many repetitions of the straight leg raise as possible within a 30 s, with the number of repetitions recorded as the outcome measure.

#### 2.3.6. Countermovement Jump (CMJ)

The countermovement jump (CMJ) was conducted using the fitness assessment tool (Afascan 100, Scanleader Technologies. Co Ltd., Santa Clara, CA, USA). Before the jump test, participants underwent a brief warm-up and stretching routine. Subsequently, participants followed the instructions provided by the device and stood on the force plate. During the CMJ, participants initiated a short ‘countermovement’ or ‘pre-stretch’ action before take-off. While in the air, participants needed to maintain extension in the hip, knee, and ankle joints to prevent any additional flight time achieved by bending their legs. Participants were instructed to jump as high as possible while attempting to land in the same position as they took off. Each participant performed the vertical jump test three times, with the best result recorded as the CMJ score.

#### 2.3.7. Balance

The balance test was conducted using the fitness assessment tool (Afascan 100, Scanleader Technologies. Co Ltd., Santa Clara, CA, USA). Participants wore sensors on both legs and stood on the force plate with one foot on each side. After a countdown, participants performed 30 s of single-leg standing with both eyes open and closed, first with their dominant foot and then with their non-dominant foot. During the test, the raised foot was positioned beside the knee of the standing leg. The performance was assessed based on the average gravity deviation (mm) detected by the force plate. This measurement was used to calculate the participant’s balance and deviation rates, and the instrument automatically computed the overall balance scores, balance rate, and shift. This specific test is called the Eyes Closed Single Leg Balance Test.

#### 2.3.8. Anaerobic Ability

Following a 10 min low-intensity running warm-up, the Running-based Anaerobic Sprint Test (RAST) was conducted according to the protocol described by Zagatto et al. [24]. The RAST consisted of six 35 m sprints at maximum speeds with intervals of 10 s for recovery between races. The time to complete each 35 m sprint was recorded to determine the average, maximum, and minimum power and fatigue index (FI). In watts (W), power for each sprint was calculated through the product of body mass in kilograms, and the distance (35 m) was raised to the second power. Afterward, this result was divided by the sprint time in seconds and raised to the third power as follows: peak power = body mass (kg) × distance (m)^2^/time (s)^3^. The highest power value produced in the sprints was considered the peak power. FI = (Maximum power − Minimum power) ÷ Total time for the six sprints.

#### 2.3.9. Aerobic Performance

The warm-up and the Yo-Yo intermittent recovery test level 1 (Yo-Yo IR1 test) were conducted indoors, within the confines of a sports hall, on a specifically marked 20 m running track, following the detailed protocol outlined by Bangsbo et al. [25]. The test involved a series of repeated 2 × 20 m sprints, requiring participants to run between a designated starting line, a turning point, and a finish line. The speed of these sprints progressively increased and was precisely controlled using auditory signals emitted by an audio system. Following each 2 × 20 m sprint, participants were given 10 s for active recovery within a demarcated 5 × 2 m area, indicated by cones positioned behind the starting and finishing lines. In cases where a participant failed to cross the finish line before the final auditory signal, a warning was issued. If a participant subsequently failed to reach the finish line before the acoustic signal for a second time, the distance covered at that point was meticulously recorded and served as the conclusive test result.

### 2.4. Statistical Analyses

All data were reported as the mean ± SD. The normality of the data was evaluated using the Shapiro–Wilk test. Comparisons for all performance variables between pre- and post-test were analyzed using a 2 × 2 (group × time) repeated measures two-way analysis of variance (ANOVA). All statistical analyses were conducted using SPSS version 10.0 for Windows (IBM SPSS, Armonk, NY, USA). A *p*-value of 0.05 was considered significant.

## 3. Results

**Figure 2:**
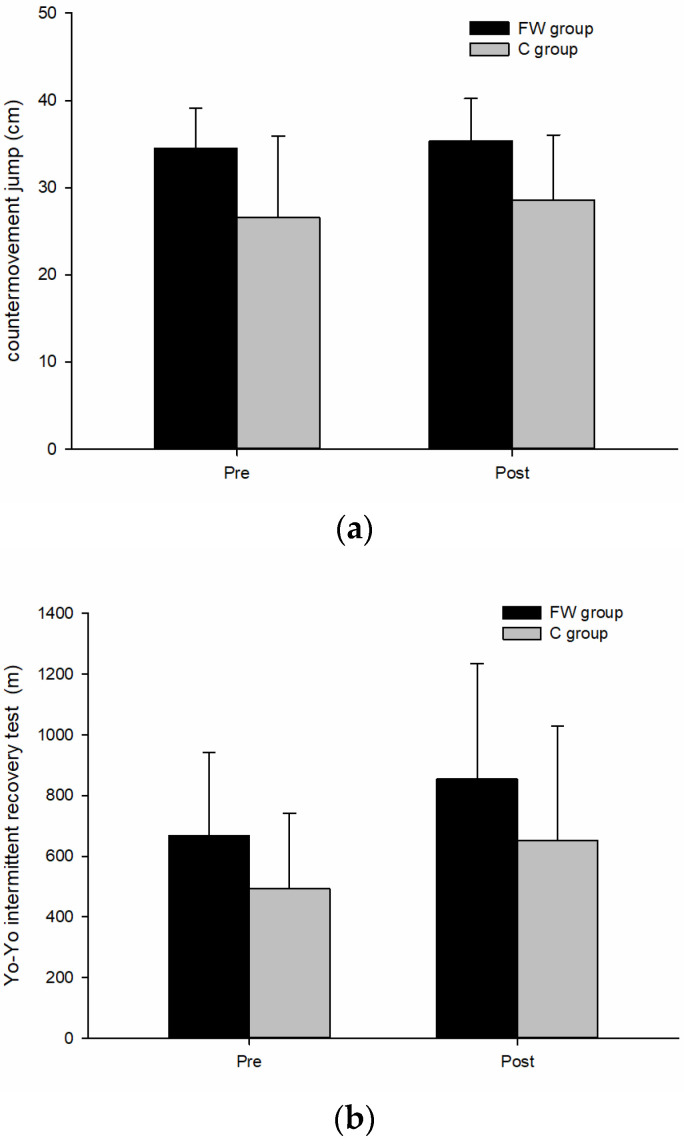

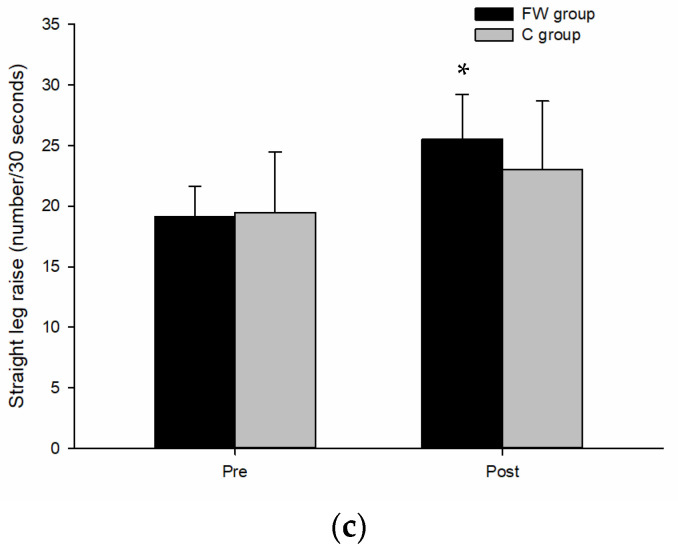
(**a**) Countermovement jump; (**b**) Yo-Yo intermittent recovery test; (**c**) Straight let raise test before (Pre) and after (Post) 24-weeks training. FW = free weight resistance training group; (**c**) = control group. * Significant difference from corresponding time points between pre- and post-test (*p* < 0.05).

The primary findings of this study revealed that youth canoe/kayak athletes, following 24 weeks of free-weight resistance training, showed a significant increase in body weight in the FW group (*p* < 0.05), as indicated in Table 1. However, no significant differences between groups were observed in body fat percentage, skeletal muscle mass, or body fat mass. Regarding upper-body isometric muscle strength and medicine ball throw power, as presented in Table 2, there were no significant differences observed in the performance of isometric muscle strength tests, including seal row, bench press, and mid-thigh pull, between the FW group and the control group who underwent bodyweight training. This study evaluated upper-body power using the medicine ball throw test. However, no significant differences were observed between the FW and control groups in chest push, overhead front throw, and overhead back throw performances. In the anaerobic ability test of the RAST, as shown in Table 3, no significant differences were observed between the two groups in peak power, mean power, and fatigue index performance. Similarly, as presented in Table 4, no significant differences were observed between the two groups in the balance test. Regarding various athletic performance tests, including vertical jump and aerobic capacity tests, as depicted in Figure 2a,b, no significant differences were observed between the two groups. However, in terms of core muscle strength performance, as illustrated in Figure 2c, the FW group demonstrated a more significant improvement in the number of straight leg raise repetitions after 24 weeks of training compared to the control group who underwent bodyweight training (25.53 ± 3.70 vs. 23.00 ± 5.69, *p* < 0.05).

## 4. Discussion

Given the relatively low prevalence of free weight resistance training among adolescents in Taiwan, this study is the first study comparing the effects of free weight resistance and bodyweight training on the athletic performance of adolescent canoe/kayak athletes. The primary finding of this study was that after 24 weeks of training, the free-weight resistance training group exhibited a significant increase in body weight and notable improvements in core muscle strength compared to the bodyweight training control group. However, the two groups observed no significant differences between upper-body isometric muscle strength, power, balance, and anaerobic/aerobic performance.

Resistance training has been shown to induce various beneficial effects on athletes’ bodies, with one of the most prominent being the increase in muscle mass, as indicated by muscle cross-sectional area [26]. Consistent with previous research findings [13,27], the current study also found that athletes in the free-weight resistance training group experienced a more significant increase in body weight after 24 weeks of training. Different forms of resistance training can lead to immediate and prolonged hormonal alterations, particularly involving insulin-like growth factor (IGF-1), testosterone, and growth hormone, which are crucial in facilitating hypertrophic signaling responses [28,29,30]. Although muscle mass did not increase significantly in the free-weight resistance training group in this study, it is possible that free-weight resistance training still produces more favorable metabolic synthesis effects compared to bodyweight training. The study conducted by Kukic et al. [31] indicated a significant positive correlation between kayak single-stroke maximal power (SSKTpmax) and body mass, BMI, and skeletal muscle mass index (SMMI). These research findings suggest that increased body weight may potentially contribute to better force and power outputs of the kayak stroke. 

Another significant finding of this study was that after 24 weeks of free weight resistance training, athletes in the FW group showed a substantial improvement in the number of leg raises during the 30 s straight leg raise test compared to the control group. This test primarily evaluates the core strength of the athletes. The term “core” has been used to describe the lumbopelvic hip complex and the surrounding musculature, as outlined by Bergmark [32]. The core includes various muscle groups, such as the rectus abdominis and the internal and external obliques. Functionally, these muscles are the central hub for force transmission through kinetic chains to the extremities [33]. Core muscles are categorized into local stabilizers and global force generators. Weak core muscles may lead to instability [34]. Therefore, enhancing the strength and stability of these core muscles becomes a crucial element in the training regimen for competitive athletes [35]. Previous research has indicated that free-weight exercises increase joint stabilization demands compared to stable machines [36,37]. Consistent with these findings, our study suggests that training with free-weight resistance exercises may be more effective in improving core strength in adolescent athletes compared to bodyweight exercises.

In this study, the FW and the control groups showed significant improvements in upper-body strength and power performance, as assessed by isometric muscle strength tests and medicine ball throws, respectively, after 24 weeks of training. However, the magnitude of improvement did not significantly differ between the two training groups. There are no specific age requirements for young individuals to engage in resistance training. National governing bodies overseeing strength and conditioning advocate for resistance training for children once they are physically and mentally prepared for sports participation. This readiness is determined by their ability to follow instructions, which is crucial for ensuring safety [6]. In previous research, Rodriguez-Rosell et al. [38] implemented low-load, high-velocity resistance training with the same duration and frequency among youth soccer players. They found that all age groups (U13, U15, and U17) showed significant improvements in strength, jump, and sprint assessments, although the degree of improvement decreased with increasing chronological age. Various free-weight training techniques have produced favorable adaptations in strength and power among young individuals. These techniques include heavy strength training, weightlifting, peak-power training, and combinations [39,40].

The correlation between training intensities (%RM) and improvements in maximal strength and motor skill performance in youth populations has been well documented [10]. Strength training typically involves high-load resistance training relative to an individual’s maximal strength (>80% 1RM), utilizing two to four sets at low-repetition ranges (<6) [41]. Moreover, recent research suggests an effective intensity range to improve strength in youth athletes is under 80–89% 1RM [12]. As a result, there may not have been significant differences in the intensity of the loads used between the FW and the control groups, as both groups mainly focused on learning proper movement techniques. This could contribute to the lack of significant differences in strength and power improvements between the FW and C groups in this study.

After 24 weeks of training, the two groups showed no significant differences in anaerobic capacity, as measured by the Running-based Anaerobic Sprint Test (RAST), and aerobic capacity, as measured by the Yo-Yo test. This lack of difference may be attributed to the fact that the control group athletes, to maintain their original training conditions, incorporated running and high-intensity interval training into their daily routines and bodyweight training. Interestingly, previous studies have suggested a strong positive correlation between peak power in the RAST and vertical jump performance in teenage futsal athletes [42] and professional basketball players [43]. Similarly, our study found a significant correlation between vertical jump height and peak power (r = 0.703, *p* < 0.01). Moreover, further analysis revealed an even higher correlation between vertical jump height and peak power per weight (r = 0.838, *p* < 0.01). From a physiological standpoint, it is reasonable to assume a significant correlation between the vertical jump test and RAST. This assertion is supported by the shared utilization of the primary metabolic pathway, ATP-CP, during both tests. Additionally, the stretching–shortening cycle contributes to this correlation by enhancing muscular force through the combination of mechanical and elastic forces generated during muscle stretching [42].

Balance is a crucial ability in canoeing and kayaking, where athletes must paddle on unstable water surfaces while coordinating their movements and exerting force throughout their entire body. In our study, balance ability did not differ significantly between the two groups. This lack of difference may be attributed to the testing method, which involved standing on a force plate for both closed-eye and open-eye single-leg balance measurements. The assessment evaluated participants’ balance ability based on the displacement of force measured by the force plate under their feet. However, the paddling motion in canoeing and kayaking occurs while seated, and the standing balance test implemented in our study may not accurately reflect the balance ability of canoeing and kayaking athletes. Previous research has attempted to investigate the relationship between upper-body and lower-body stability and mobility as assessed by the Y-balance test and kayak performance. However, statistically significant correlations were not found [2]. In future studies, it would be worthwhile to explore how to appropriately assess the balance abilities of kayak athletes, as this remains an important topic for further investigation.

Distinguishing between the growth of strength and power in athletes, whether it arises from training factors or maturation and developmental muscle strength growth and neuromuscular adaptability, may interfere with the assessment of training effects, potentially leading to erroneous conclusions regarding the effectiveness of strength training for adolescent athletes [13]. Nonetheless, this study has limitations, as the random grouping resulted in an uneven number of genders between the two groups during the pre-test, creating initial differences in anthropometry, body composition, and performance. Additionally, the growth factors of adolescent participants during the 24 weeks were also considered. All adolescent participants were novices to resistance training and required more time to adapt to techniques and master the proper form and intensity of free weight training.

## 5. Conclusions

This study compared the differences between free-weight and bodyweight resistance training on sports performance in adolescent canoe/kayak athletes. The findings revealed that, aside from yielding superior results in enhancing core muscle strength compared to bodyweight training, no significant differences were observed between the two groups regarding other related indicators such as body composition, muscular strength, and physical fitness. Therefore, based on the results of this study, it cannot be conclusively stated that 24 weeks of free-weight training significantly enhances the performance of youth canoe/kayak athletes. Instead, it can only be supported that it contributes to increased core muscle strength. However, the practical implications of this study lie in its rarity as one of the few investigations applying free-weight resistance training to youth canoe/kayak athletes. Additionally, it was noted that a 24-week training duration might be insufficient for novice participants in resistance training. Future research should consider incorporating an adaptation period or a learning phase for movements before training, thereby potentially enhancing the efficacy of free-weight resistance training in increasing strength.

## Figures and Tables

**Table 1:** Participants’ body composition before and after 24 weeks of training.

	FW Group		C Group	
Variables	Pre	Post	Pre	Post
Height (cm)	164.88 ± 5.55	165.87 ± 5.89	161.44 ± 6.76	161.90 ± 6.56
Body mass (kg)	54.46 ± 5.57	55.95 ± 4.96 *	59.16 ± 10.39	58.00 ± 10.39
Body fat (%)	14.70 ± 8.27	14.32 ± 6.48	24.43 ± 8.08	21.53 ± 6.77
Skeletal muscle mass (kg)	25.62 ± 3.15	26.71 ± 3.29	24.42 ± 4.55	25.12 ± 4.55
Body fat mass (kg)	8.64 ± 4.98	8.10 ± 3.81	14.3 ± 6.95	12.58 ± 5.99
Body mass index	20.21 ± 2.44	20.39 ± 1.84	22.35 ± 2.63	21.96 ± 2.56

* *p* < 0.5 Significant difference between pre- and post-test.

**Table 2:** Upper-body isometric muscle strength and power before and after 24 weeks of training.

	FW Group		C Group	
Variables	Pre	Post	Pre	Post
Isometric muscle strength				
Seal row (N)	1030.16 ± 105.03	1057.73 ± 137.86	1022.96 ± 297.29	1028.94 ± 221.81
Bench press (N)	575.73 ± 103.83	778.62 ± 148.82	602.04 ± 180.07	839.42 ± 247.62
Mid-thigh pull (N)	1667.82 ± 409.30	1769.47 ± 338.09	1681.66 ± 543.28	1727.98 ± 536.86
Medicine ball throw				
Chest push (cm)	501.44 ± 84.82	484.31 ± 53.00	452.44 ± 111.75	470.25 ± 67.77
Overhead front throw (cm)	505.17 ± 90.34	473.16 ± 64.57	488.45 ± 140.01	509.81 ± 105.45
Overhead back throw (cm)	639.20 ± 203.71	523.24 ± 80.52	583.72 ± 197.72	528.95 ± 108.97

**Table 3:** Anaerobic ability before and after 24 weeks of training.

	FW Group		C Group	
Variables	Pre	Post	Pre	Post
Peak power (W)	397.73 ± 84.91	427.34 ± 101.11	336.83 ± 146.35	343.42 ± 128.52
Mean power (W)	313.75 ± 70.60	336.13 ± 86.83	336.13 ± 86.83	260.30 ± 94.07
Peak power per weight (W·kg^−1)^	7.35 ± 1.65	7.64 ± 1.75	5.73 ± 2.06	5.97 ± 1.82
Mean power per weight (W·kg^−1^)	5.78 ± 1.31	5.99 ± 1.46	4.33 ± 1.90	4.59 ± 1.50
Fatigue index (%)	4.13 ± 1.69	4.66 ± 1.39	3.51 ± 1.91	3.62 ± 2.07

**Table 4:** Single-leg balance test before and after 24 weeks of training.

	FW Group		C Group	
Variables	Pre	Post	Pre	Post
Open eyes and stand on right foot				
Scores	48.62 ± 16.85	48.91 ± 12.25	50.72 ± 9.82	52.89 ± 7.99
Average center of mass deviation (mm)	13.91 ± 4.75	15.22 ± 4.05	14.11 ± 3.94	12.97 ± 2.67
balance rate (%)	16.59 ± 10.10	14.31 ± 8.07	16.56 ± 8.08	15.57 ± 5.76
shift	0.78 ± 0.69	0.58 ± 0.42	0.45 ± 0.20	0.39 ± 0.18
Open eyes and stand on left foot				
Scores	48.46 ± 13.91	48.29 ± 16.44	51.06 ± 9.76	51.97 ± 9.44
Average center of mass deviation (mm)	14.33 ± 5.10	14.89 ± 5.83	13.45 ± 2.72	13.08 ± 2.15
balance rate (%)	15.11 ± 7.02	16.53 ± 9.66	15.47 ± 7.01	16.53 ± 9.66
shift	0.65 ± 1.07	0.55 ± 0.49	0.38 ± 0.19	0.36 ± 0.11
Close eyes and stand on right foot				
Scores	21.36 ± 13.72	25.67 ± 11.04	32.44 ± 8.64	36.89 ± 6.72
Average center of mass deviation (mm)	32.45 ± 17.06	29.29 ± 14.29	21.33 ± 8.46	18.47 ± 5.02
balance rate (%)	5.60 ± 5.33	6.50 ± 3.90	8.47 ± 3.45	9.22 ± 2.44
shift	4.80 ± 5.93	5.07 ± 6.44	2.58 ± 2.92	1.59 ± 1.57
Close eyes and stand on left foot				
Scores	25.38 ± 11.36	27.91 ± 10.81	31.28 ± 8.14	35.36 ± 5.11
Average center of mass deviation (mm)	26.87 ± 8.73	25.47 ± 11.92	20.83 ± 4.70	18.61 ± 3.58
balance rate (%)	6.02 ± 3.51	6.91 ± 3.63	8.21 ± 3.03	9.64 ± 4.18
shift	2.51 ± 2.05	3.27 ± 3.76	1.58 ± 1.02	1.26 ± 1.33

## Data Availability

The data presented in this study are available on request from the corresponding author. The data are not publicly available due to ethical restrictions.
